# Cis-urocanic acid suppresses UV-B-induced interleukin-6 and -8 secretion and cytotoxicity in human corneal and conjunctival epithelial cells in vitro

**Published:** 2009-09-08

**Authors:** J. Viiri, H.M. Jauhonen, A. Kauppinen, T. Ryhänen, T. Paimela, J. Hyttinen, I. Sorri, J.K. Laihia, L. Leino, K. Kaarniranta

**Affiliations:** 1 Department of Ophthalmology, Institute of Clinical Medicine, University of Kuopio, Kuopio, Finland; 2 Department of Ophthalmology, Kuopio University Hospital, Kuopio, Finland; 3 Department of Neurology and Neurosciences, Institute of Clinical Medicine, University of Kuopio, Kuopio, Finland; 4 BioCis Pharma Ltd, Turku, Finland

## Abstract

**Purpose:**

Urocanic acid (UCA) is a major ultraviolet (UV)-absorbing endogenous chromophore in the epidermis and is also an efficacious immunosuppressant. The anti-inflammatory and cytoprotective effects of cis-UCA were studied in ocular surface cell cultures exposed to UV-B irradiation.

**Methods:**

Human corneal epithelial cells (HCE-2) and human conjunctival epithelial cells (HCECs) were incubated with 10, 100, 1,000, and 5,000 μg/ml cis-UCA with and without a single UV-B irradiation dose. The concentrations of IL-1β, IL-6, IL-8, and TNF-α in the culture medium and caspase-3 activity in the cell extract sampled were measured by enzyme-linked immunosorbent assay (ELISA). Cell viability was measured by the colorimetric MTT (3-(4,5-dimethyldiazol- 2-yl)-2,5-diphenyltetrazolium bromide) assay.

**Results:**

UV-B irradiation multiplied interleukin IL-6 and IL-8 secretion levels in HCE-2 cells and HCECs as analyzed with ELISA. Cell viability as measured by the MTT assay declined by 30%–50% in HCE-2 cells and by 20%–40% in HCECs after UV-B irradiation. Moreover, UV-B increased caspase-3 activity in both cell types as analyzed with ELISA. Treatment with 100 μg/ml cis-UCA completely suppressed IL-6 and IL-8 secretion, decreased caspase-3 activity, and improved cell viability against UV-B irradiation. No significant effects on IL-6 or IL-8 secretion, caspase-3 activity, or viability of the non-irradiated cells were observed with 100 μg/ml cis-UCA in both cell types. The 5,000 μg/ml concentration was toxic.

**Conclusions:**

These findings indicate that cis-UCA may represent a promising anti-inflammatory and cytoprotective treatment option to suppress UV-B-induced inflammation and cellular damage in human corneal and conjunctival epithelial cells.

## Introduction

Urocanic acid (UCA) is a major ultraviolet (UV)-absorbing chromophore in the epidermis, and it has been proposed to function as a regulator of UV-induced damage in photoimmunology [1]. The cis-UCA, formed from trans-UCA upon UV-B exposure, has been implicated in the down-regulation of hypersensitivity reactions [2,3] in the actions of epidermal antigen-presenting cells [4,5], the activation of neutrophils [6,7], and the prolonged survival of organ transplants [8], but the mechanisms of action still remain to be resolved.

Ocular surface cells including corneal and conjunctival cells are frequently exposed to UV radiation, which may evoke epithelial damage, cell death, and inflammation [9,10]. Photokeratoconjunctivitis, pinguecula, pterygium, nodular band keratopathies, and epidermoid carcinoma are believed to be the result of exposure to intense UV radiation of the ocular surface [11]. The dry eye syndrome is the most common reason for ocular surface inflammation [12,13]. Dry eye is associated with irritation of the ocular surface and increased risk of secondary infections [14]. In the multifactorial dry eye syndrome, there appears to be increased production of proinflammatory cytokines such as interleukin-1 (IL-1), interleukin-6 (IL-6), interleukin-8 (IL-8), tumor necrosis factor-alpha (TNF-α), and proteolytic enzymes. These cytokines are released not only from the ocular surface and glandular epithelial cells but also from the infiltrating inflammatory cells [15-19]. In this study, we have investigated the role of cis-UCA in IL-1β, IL-6, IL-8, and TNF-α immune response and cytotoxicity that are important factors of inflammatory reactions in ocular cell types. We show that cis-UCA suppresses UV-B-induced inflammation and cellular damage in human corneal and conjunctival epithelial cells.

## Methods

### Cell culture

Human corneal epithelial cells (HCE-2) and human conjunctival epithelial cells (HCECs) were obtained from American Type Culture Collection (Manassas, VA). The HCE-2 cells were cultured in Keratinocyte-SFM medium with supplements (25 mg bovine pituitary extract and 2.5 µg human recombinant epidermal growth factor; Gibco Invitrogen, Paisley, UK) including 10% fetal bovine serum  (Hyclone, Logan, UT), 100 U/ml penicillin, 100 µg/ml streptomycin (Gibco Invitrogen), and 0.005 mg/ml insulin (Sigma-Aldrich, St. Louis, MO). HCECs were cultured in Medium 199 (Gibco Invitrogen) supplemented with serum and antibiotics as described above. Confluent cultures of both cell lines were treated with concentrations of 10, 100, 1,000, and 5,000 µg/ml cis-UCA (BioCis Pharma, Turku, Finland) and exposed to a UV-B irradiation dose of 153 mJ/cm^2^ (four TL 20W/12 tubes, Philips, Eindhoven, The Netherlands) in 12 well plates. The irradiation was performed at room temperature for 1 min using a source-to-target distance of 30 cm.

### Enzyme-linked immunosorbent assay

The concentrations of IL-1β, IL-6, IL-8, and TNF-α in the culture medium and caspase-3 activity in the cell extract sampled at 24 h, 48 h, or 72 h were measured by enzyme-linked immunosorbent assay (ELISA) using OptEIA™ sets from BD PharMingen (San Diego, CA) according to the manufacturer's instructions.

### MTT assay

Cell viability was assessed at 24 h, 48 h, or 72 h after the start of the treatments by the 3-(4,5-dimethylthiazol-2-yl)-2,5-diphenyl tetrazolium bromide (MTT) test (Sigma-Aldrich) in 12 well plates. Briefly, 25 μl of MTT solution (10 mg/ml in PBS) was added to 500 µl of culture medium and incubated for 1.5 h at 37 °C. Then, 525 μl of MTT lysis buffer (20% SDS, 50% dimethylformamide, pH 4.7) was added to the wells to induce cellular lysis. The colorimetric assay is based on the ability of viable cells to metabolize MTT. The absorbance was measured at 550 nm in a microplate reader (Bio-Rad Laboratories, Hercules, CA).

### HPLC analysis

Culture medium samples taken at the end of the experiment were assayed for cis- and trans-UCA. Proteins in the 200 μl medium samples were precipitated with 400 µl of trichloroacetic acid on ice for 10 min, centrifuged (12,800× g, 10 min, 4 °C), and analyzed with HPLC using the Agilent 1100 UV detection system (Agilent Technologies, Santa Clara CA) at 268 nm.

### Statistical analysis

The statistical significance was analyzed with SPSS for Windows software (v. 11.5; SPSS, Chicago, IL) using Mann–Whitney *U*-test. p-values below 0.05 were considered significant.

## Results

**Figure 1:**
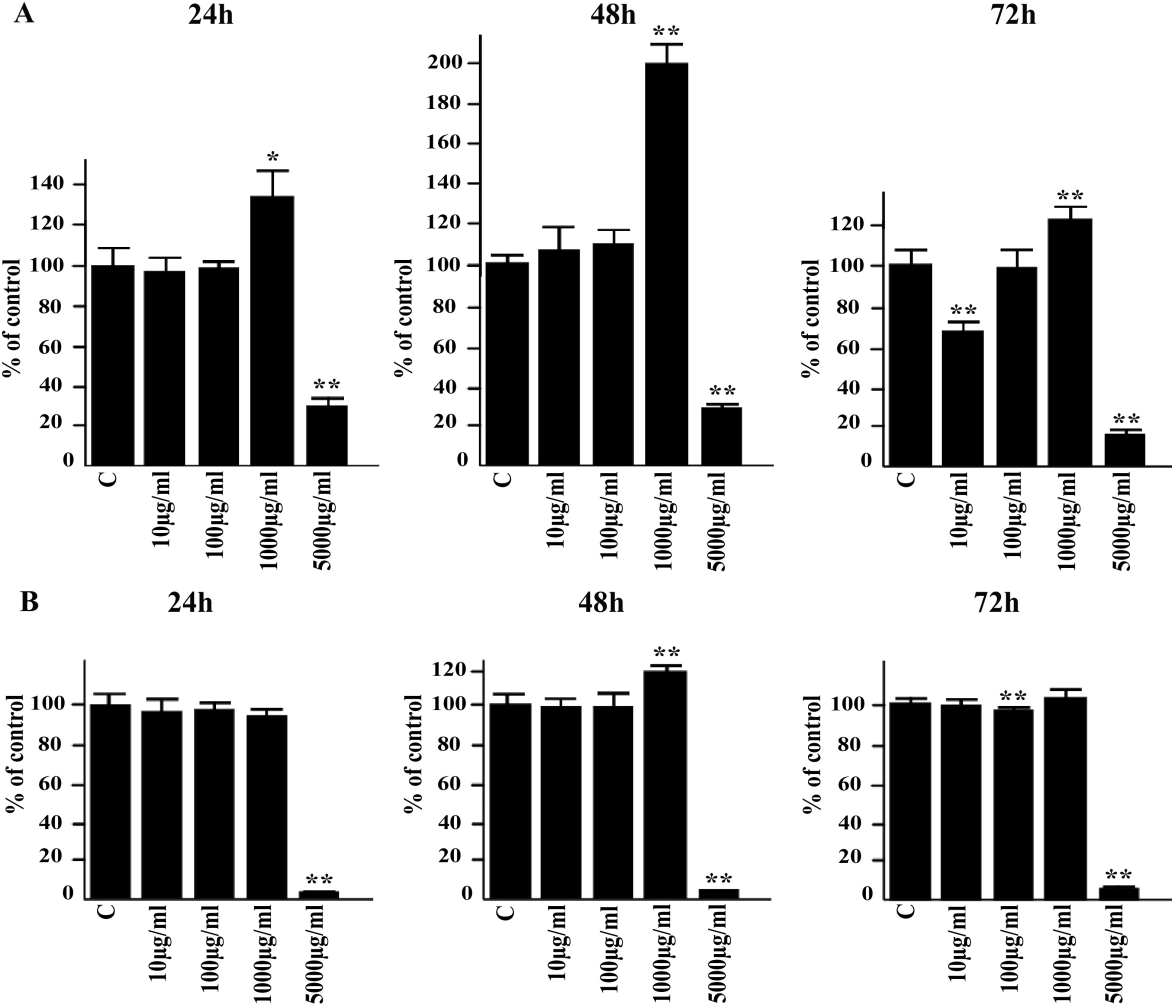
Effect of cis-UCA on IL-6 secretion. The HCE-2 cells (**A**) and HCECs (**B**) were either untreated (C) or exposed to different concentrations of cis-UCA for 24, 48, or 72 h. For statistical analysis, cis-UCA treated samples were compared with C samples. An asterisk indicates p<0.05, and a double asterisk denotes p<0.001 (n=6 dishes).

**Figure 2:**
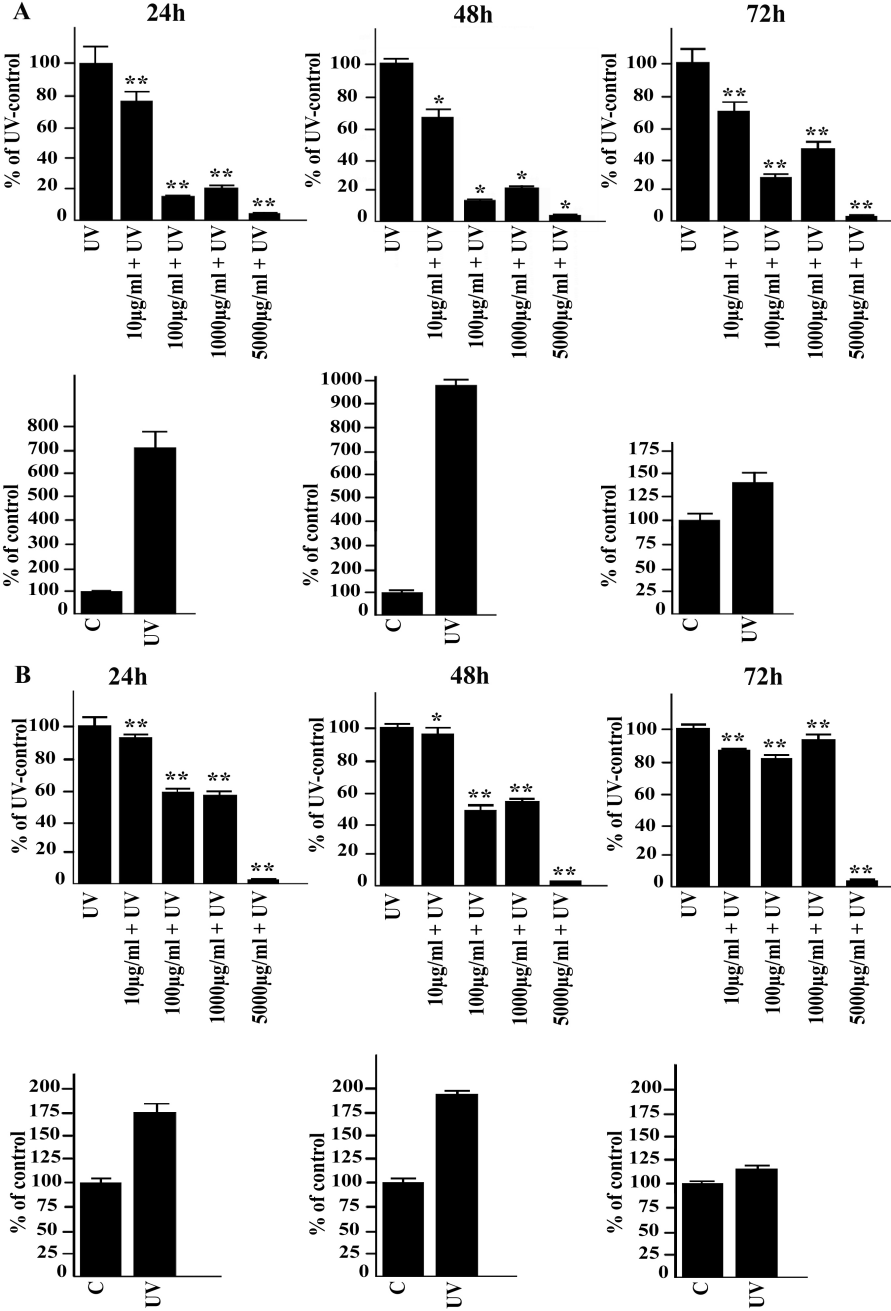
Effect of cis-UCA on UV-irradiation-induced IL-6 secretion. The HCE-2 cells (**A**) and HCECs (**B**) were non-irradiated (C) or UV-irradiated (UV; lower panels), or exposed to different concentrations of cis-UCA or UV irradiated and treated with cis-UCA for 24, 48, or 72 h (upper panels). For statistical analysis, cis-UCA+UV samples were compared with UV samples. An asterisk indicates p<0.05, and a double asterisk denotes p<0.001 (n=6 dishes).

In non-irradiated cells, the 10 and 100 µg/ml concentrations of cis-UCA had only a negligible effect on IL-6 production while treatment with 1,000 µg/ml of cis-UCA evoked a mild but significant elevation on IL-6 levels in both cell types. A major decrease in IL-6 secretion was observed with 5,000 µg/ml cis-UCA in all time points (Figure 1A,B). Exposure of the HCE-2 cells and HCECs to UV-B irradiation induced a maximum sevenfold to ninefold increase and twofold increase in the measured IL-6 concentrations, respectively. Treatment of both cell lines with cis-UCA at all studied concentrations significantly decreased the UV-B-induced IL-6 secretion (Figure 2A,B). The 100 µg/ml concentration of cis-UCA completely restored the UV-B-induced increase in IL-6 secretion back to the level detected in the non-irradiated cells after the 24-, 48-, and 72-h follow-up. IL-1β could not be detected in the control, cis-UCA-treated, or UV-B-irradiated cells (data not shown).

**Figure 3:**
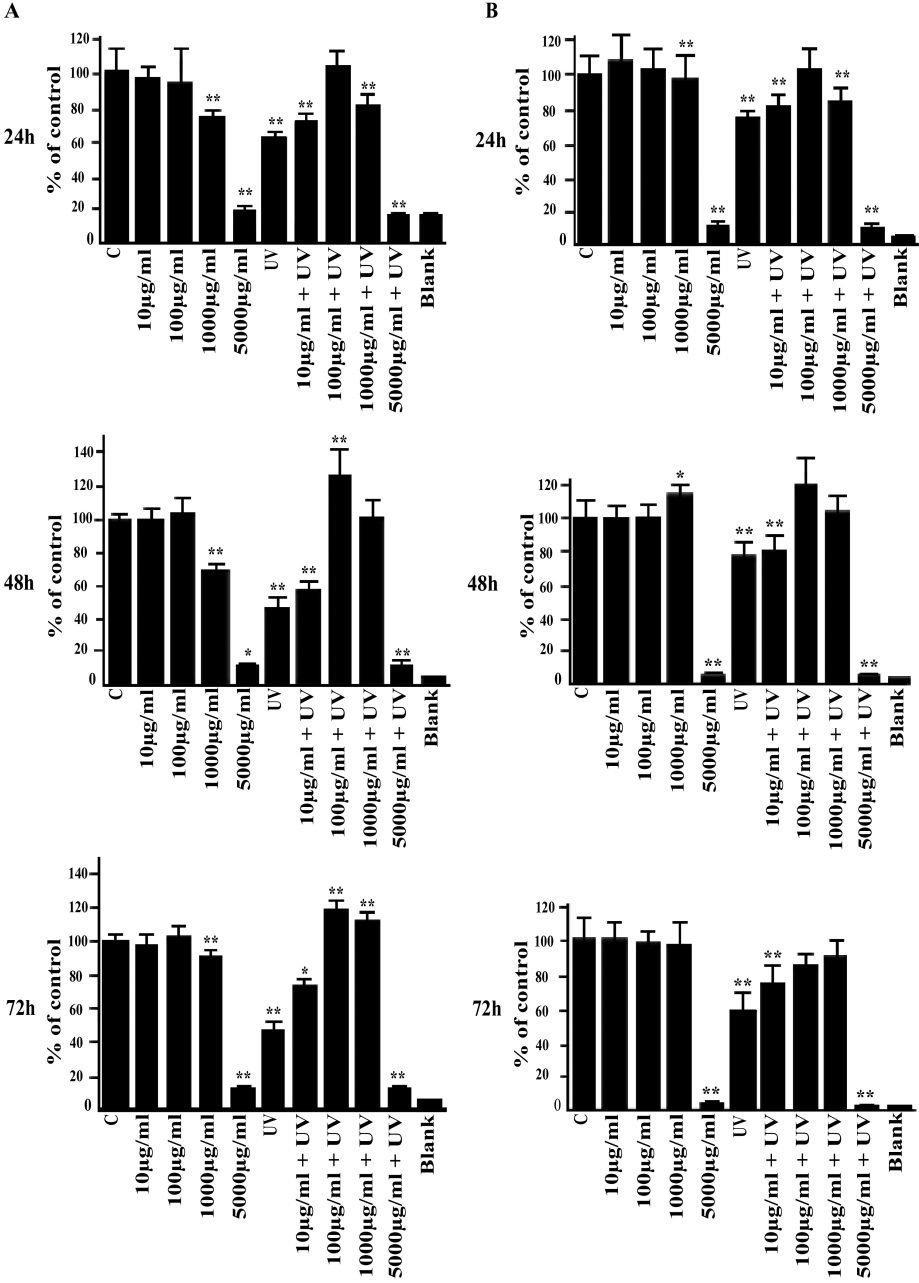
Effects of cis-UCA and UV irradiation on viability.  The non-irradiated (C) and UV-irradiated (UV) HCE-2 cells (**A**) and HCECs (**B**) were exposed to different concentrations of cis-UCA for up to 72 h. For statistical analysis, cis-UCA samples and cis-UCA+UV samples were compared with C samples. An asterisk indicates p<0.05, and a double asterisk denotes p<0.001 (n=6 dishes).

Analysis of cell viability by the MTT assay revealed no significant effect in response to 10 and 100 µg/ml cis-UCA in the non-irradiated cells during the 24-, 48-, and 72-h follow-up (Figure 3A,B). The 1,000 µg/ml concentration evoked a slight decrease in viability in the HCE-2 cells but not in HCECs while exposure to 5,000 µg/ml cis-UCA caused a clear reduction of MTT metabolism, evidence of increased cytotoxicity in both cell types (Figure 3A,B). Exposure of the cells to UV-B decreased the viability by 20%–50% . Interestingly, the 100 µg/ml concentrations of cis-UCA restored the metabolic activity of the UV-irradiated cells to the level of the non-irradiated cells in all time points (Figure 3A,B), pointing to the presence of a cytoprotective effect of cis-UCA.

**Figure 4:**
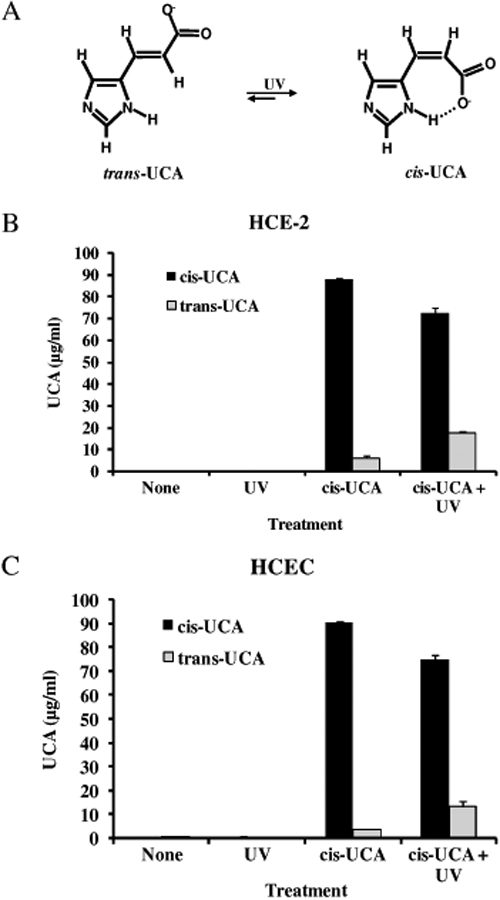
Photoisomerization of UCA. UV excitation of one isomer leads to the formation of the other isomer (**A**). The concentrations of UCA isomers is measured in the cell culture medium of HCE-2 cells (**B**) and HCECs (**C**) treated with 100 µg/ml cis-UCA for 24 h with or without UV irradiation.

In the functional assays with HCE-2 cells and HCECs, cis-UCA was present in the culture medium at the time of irradiation and during the 24 h recovery period. Since the UCA isomers absorb in the UV-B wavelength region and can photoisomerize to each other (Figure 4A), it was investigated whether photoisomerization had actually taken place in the experiments and could have affected the biological response. A total of 16 medium samples from the assays were subjected to HPLC analysis. The mean concentration of cis-UCA in the cis-UCA-treated (100 µg/ml) medium samples was 87.8 µg/ml (HCE-2) and 90.5 µg/ml (HCEC). The rest of the cis-UCA had apparently been taken up by the cells. Trans-UCA was detected in the non-irradiated samples at levels of 6.4% in HCE-2 cells and 4.0% in HCECs from the total UCA (Figure 4B,C). After exposure to UV-B irradiation as shown above, the net photoisomerization to trans-UCA was 13.1% in HCE-2 cells and 11.1% in HCECs. The cis-UCA concentrations were 72.3 μg/ml in HCE-2 cells and 74.9 μg/ml in HCECs in response to UV-B (Figure 4). This level of photoisomerization was estimated to have a negligible effect on cytokine secretion and viability.

**Figure 5:**
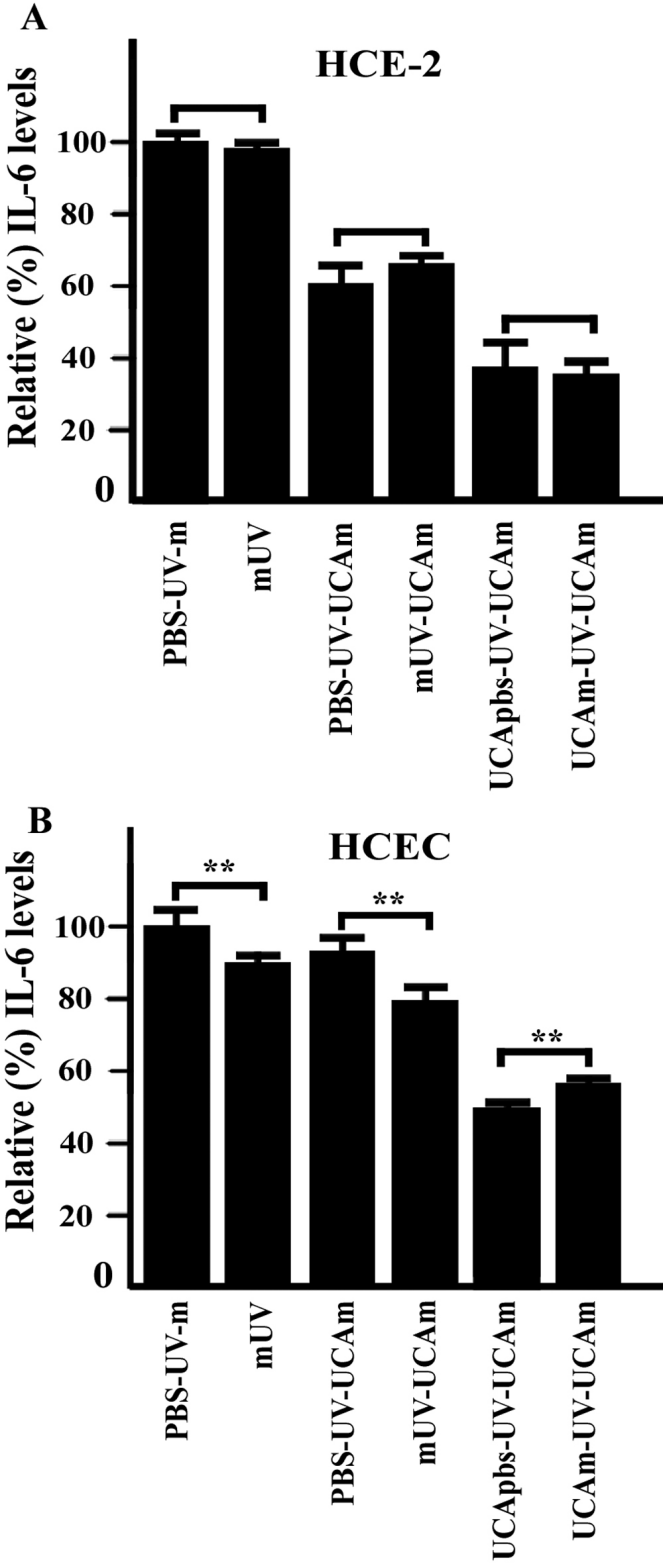
UV absorption effect of the culture medium on IL-6 secretion. The HCE-2 cells (**A**) and HCECs (**B**) were irradiated in a colorless buffer solution or in a normal culture medium prior to change of medium without or with 100 μg/ml cis-UCA and were then followed for 24 h. An asterisk indicates p<0.05, and a double asterisk denotes p<0.001 (n=6 dishes). Abbreviations: C, control; m, medium; PBS, phosphate buffered saline; UCA, urocanic acid; UV, ultraviolet.

Next, the effect of  phenol red on IL-6 secretion in response to UV-B irradiation of the cells was examined. The cells were irradiated in a colorless buffer solution or in a normal culture medium prior to change of medium without or with 100 μg/ml cis-UCA and were then followed for 24 h. As shown in Figure 5, no significant differences between the effects of the phenol red-containing medium and the colorless solution on IL-6 secretion can be seen in ocular surface cells.

**Figure 6:**
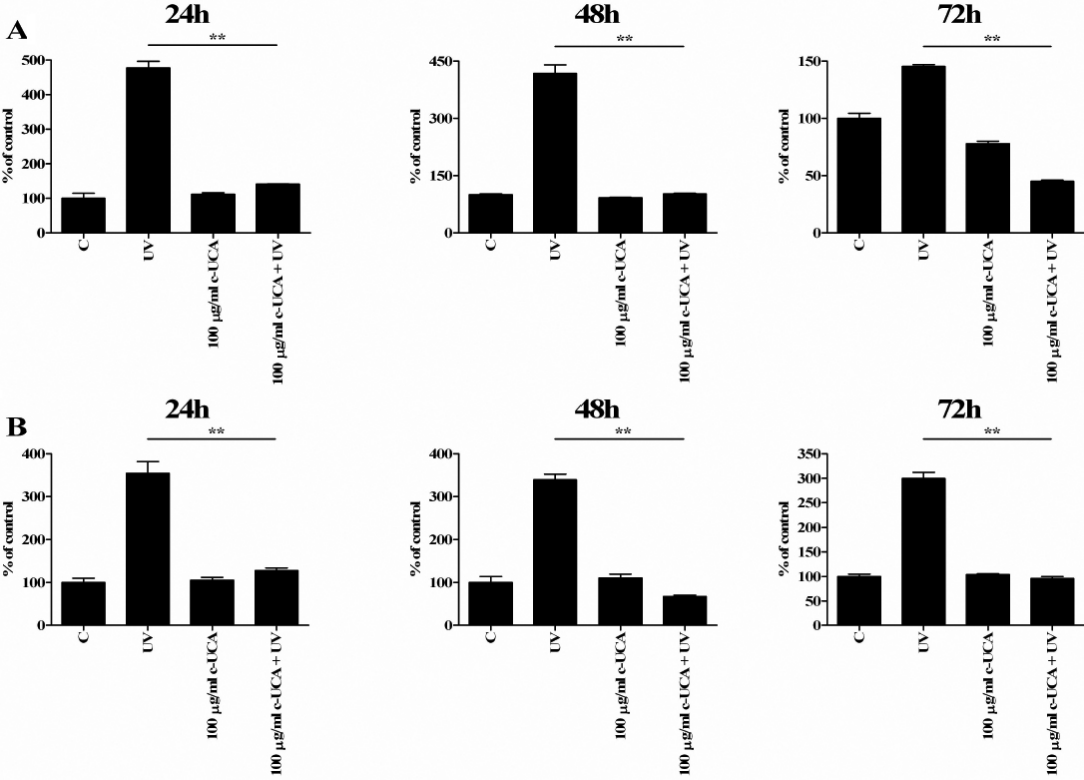
Effect of cis-UCA on UV-irradiation-induced IL-8 secretion.  The HCE-2 cells (**A**) and HCECs (**B**) were non-irradiated (C), UV-irradiated (UV), treated with 100 mg/ml cis-UCA (c-UCA), or UV-irradiated and treated with cis-UCA (100 mg/ml c-UCA+UV) for 24, 48, or 72 h. Statistical significance is shown by an asterisk (p<0.05) or a double asterisk (p<0.001; n=6 dishes). Compared samples are shown by the horizontal lines.

The 100 µg/ml concentration of cis-UCA completely restored the UV-B-induced increase in IL-6 secretion (Figure 2). Therefore, we wanted to analyze IL-8 and TNF-α secretion in similar conditions. Exposure of the HCE-2 cells and HCECs to UV-B irradiation induced a four- to fivefold and threefold increase in the measured IL-8 concentrations, respectively. Treatment of both cell lines with 100 µg/ml cis-UCA significantly decreased the UV-B-induced IL-8 secretion (Figure 6A,B). TNF-α could not be detected in the control, cis-UCA-treated, or UV-B-irradiated cells (data not shown).

**Figure 7:**
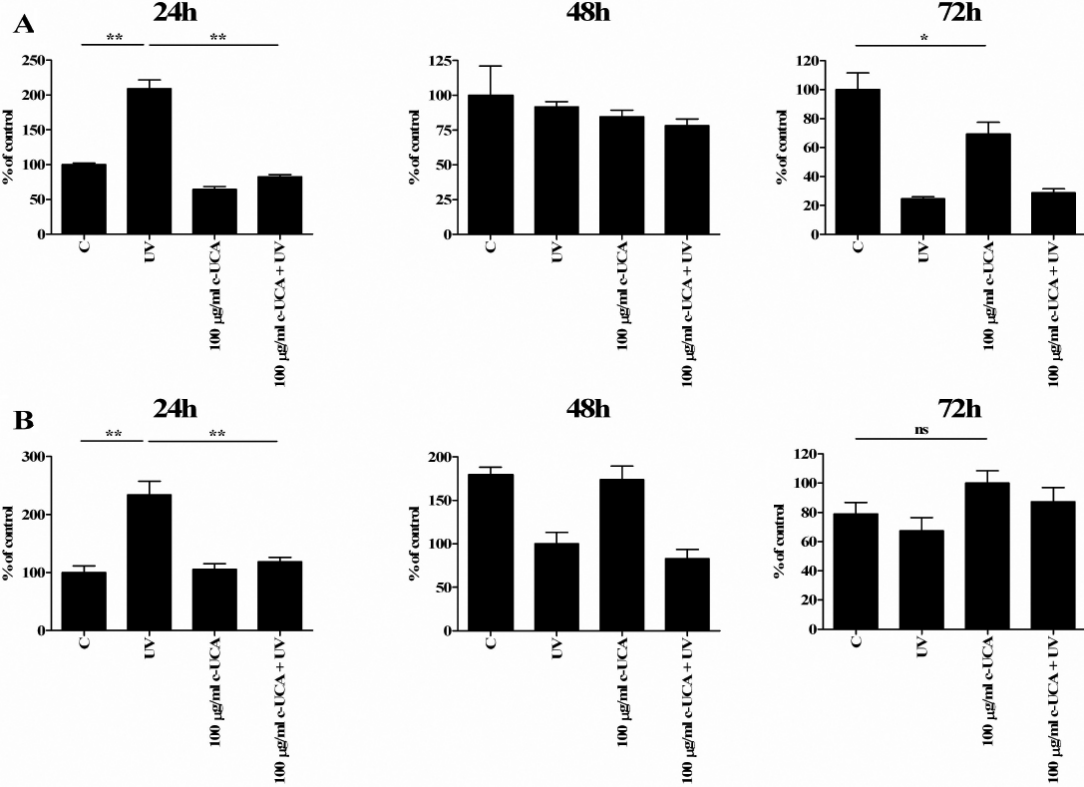
Effect of cis-UCA on UV-irradiation-induced caspase-3 activity. The HCE-2 cells (**A**) and HCECs (**B**) were non-irradiated (C), UV-irradiated (UV), treated with 100 mg/ml cis-UCA (c-UCA), or UV-irradiated and treated with cis-UCA (100 mg/ml c-UCA+UV) for 24, 48, or 72 h. Statistical significance is shown by an asterisk (p<0.05) or a double asterisk (p<0.001; n=6 dishes). Note that there is no significant difference (ns) between the control sample and the c-UCA-treated sample after the 72 h follow-up.

Since the 100 µg/ml concentration of cis-UCA restored the metabolic activity of the UV-irradiated cells (Figure 3), we finally analyzed caspase-3 activity. Treatment of both cell lines with 100 µg/ml cis-UCA significantly decreased the UV-B-induced caspase-3 activity after 24 h follow-up (Figure 7A,B). This is in line with the MTT assay results (Figure 3) and reveals anti-apoptotic effects of cis-UCA in the HCE-2 cells and HCECs.

## Discussion

Our results demonstrate that cis-UCA possesses both anti-inflammatory and cytoprotective effects against an external stress, in this case UV-B irradiation, in human corneal (HCE-2) and conjunctival epithelial (HCEC) cells. Many previous reports have described cell type dependent responses of cytokine expression after treatment with cis-UCA. Cis-UCA at concentrations of 10–100 µg/ml has been documented to increase IL-6, IL-8, and TNF-α secretion in human keratinocytes [20]. One study revealed an increased IL-10 production in activated CD4+ T cells [21] while another indicated that cis-UCA could inhibit secretion of IL-10 in T lymphocytes [22]. It has been suggested that cis-UCA may stimulate the cytokine cascade by induction of prostaglandin-E2  (PGE2) in keratinocytes [20,23] while in peripheral blood monocytes, it attenuated the PGE2- induced release of TNF-α [24]. However, there are findings that low doses of cis-UCA have not had an effect on IL-1 or IL-6 production in monocytes [24,25]. Similarly, IL-1 could not be induced by 10–100 µg/ml cis-UCA in human keratinocytes [20]. In agreement with these reports, our results indicate that low doses of cis-UCA did not affect IL-1β, IL-6, IL-8, or TNF-α secretion in human corneal and conjunctival cells. The data also show that 100 µg/ml cis-UCA significantly decreased the UV-B-induced secretion of IL-6 and IL-8 in these cells. It is of interest to note that the 1,000 µg/ml concentration of cis-UCA increased the IL-6 levels in the non-irradiated cells. However, our findings are evidence of clear anti-inflammatory activity of cis-UCA on UV-irradiated ocular surface cell types.

It is well known that UV radiation can cause cell damage and even the death of ocular surface cells [9,26]. In human and animal skin, UCA is the major UV-absorbing chromophore, but how it causes this effect is still far from clear. According to a recent view, cis-UCA may induce immune suppression via cells that express the 5-hydroxytryptamine (serotonin) receptor 2A (5-HT2A) receptor [27]. In addition to the limited signaling data, only a few studies have determined the toxicity of cis-UCA in cell cultures [28]. Our findings demonstrated good tolerability of HCE-2 cells and HCECs to low concentrations (10–1,000 µg/ml) of cis-UCA whereas the highest concentration studied (5,000 µg/ml) was clearly toxic. For comparison, a cis-UCA concentration of 3,000 µg/ml in a mildly acidic medium decreased the mitochondrial metabolic capacity by about 90% in a bladder carcinoma cell line [29]. With respect to both toxicity and biological effects, the optimal concentration in the present investigation seemed to be 100 µg/ml cis-UCA since this was able to prevent as well as to fully restore both of the measured UV-B-induced changes, i.e., the decrease in metabolic activity and the increase in IL-6 secretion, back to the level of the non-irradiated control cells. Thus, 100 µg/ml cis-UCA was optimally anti-inflammatory and cytoprotective against cellular damage caused by UV-B radiation in the present experimental setting. It should be noted that the decrease in mitochondrial metabolic activity measured by the MTT assay after UV-B irradiation may not be caused by the irradiation only because the UV-B dose induced IL-6 secretion at the nanomolar level, which can directly decrease MTT metabolism [30]. The UV-B irradiation induced caspase-3 activity in both cell types, which revealed apoptotic cell death and supported the MTT assay data. Treatment with 100 µg/ml cis-UCA also effectively prevented caspase-3 activity in the UV-B treated cells. Since elevated IL-1β secretion levels were not observed in any of the treatments, inflammasomes and caspase-1 are obviously not involved in the UV-B or cis-UCA responses. Caspase-1 is the principal caspase found in human inflammasomes that cleave the precursors of pro-inflammatory cytokines such as IL-1β to mature and active cytokines, which are subsequently secreted from cells [31].

The UV-B irradiation dose used in the study evoked a low level of photoisomerization from cis- UCA to trans-UCA. This may have caused some effect on IL-6 secretion, although we consider it unlikely. The presence of very low concentrations (<4 µg/ml) of trans-UCA in the non-irradiated samples treated with 100 µg/ml cis-UCA may reflect unintentional exposure to laboratory lighting. In previous reported studies with cis-UCA, analyses of the actual concentrations of the isomers have rarely been conducted, and often various mixtures of the cis and trans isomers have been used. It is possible that the very highest UCA concentrations (1,000 and 5,000 µg/ml) may have partially blocked the transmission of the UV-B photons in the culture medium, thereby preventing the full cell-irritating action of the irradiation. A smaller absorbing effect may have been caused by the medium ingredients, although no differential effect on IL-6 levels could be observed when using a bright buffer solution instead of the phenol red-containing colored medium. The prolonged exposure of the ocular surface to UV light can cause cellular damage. There are different stages of inflammation in the ocular surface due to alterations in the volume and composition of tear fluid [15,16,19,32,33]. Current clinical therapy for ocular surface inflammation consists of anti-inflammatory agents that do not offer any protection against UV radiation-induced damage [34]. Our in vitro findings suggest that cis-UCA may provide a safe and effective anti-inflammatory and cytoprotective treatment option against UV radiation-induced inflammation on the ocular surface.
